# Possible antidepressant mechanism of acupuncture: targeting neuroplasticity

**DOI:** 10.3389/fnins.2025.1512073

**Published:** 2025-02-13

**Authors:** Ning Xu, Yue He, Yong-Nan Wei, Lu Bai, Long Wang

**Affiliations:** ^1^Department of First Clinical Medical College, Heilongjiang University of Chinese Medicine, Harbin, China; ^2^First Affiliated Hospital, Heilongjiang University of Chinese Medicine, Harbin, China

**Keywords:** major depressive disorder, acupuncture, mechanism, neuroplasticity, complementary and alternative medicine

## Abstract

Major depressive disorder (MDD) is a highly prevalent and severely disabling psychiatric disorder that decreases quality of life and imposes substantial economic burden. Acupuncture has emerged as an effective adjunctive treatment for depression, it regulates neurotransmitters involved in mood regulation and modulates the activity of specific brain regions associated with emotional processing, as evidenced by neuroimaging and biochemical studies. Despite these insights, the precise neuroplastic mechanisms through which acupuncture exerts its antidepressant effects remain not fully elucidated. This review aims to summarize the current knowledge on acupuncture’s modulation of neuroplasticity in depression, with a focus on the neuroplasticity-based targets associated with acupuncture’s antidepressant effects. We encapsulate two decades of research into the neurobiological mechanisms underpinning the efficacy of acupuncture in treating depression. Additionally, we detail the acupoints and electroacupuncture parameters used in the treatment of depression to better serve clinical application.

## 1 Introduction

Major depressive disorder (MDD) is a highly prevalent and severely disabling psychiatric disorder (Kupfer et al., 2012). It significantly decreases quality of life and imposes substantial economic burden (Fox and Lobo, 2019; Monroe and Harkness, 2022). Although the pathogenesis of MDD has advanced considerably, it remains incompletely understood. Studies have indicated a reduction in neuroplasticity among patients with depression and in animals subjected to stress or various depression models. Brain imaging studies have shown that patients with MDD exhibit volume reductions and decreased connectivity in the prefrontal cortex (PFC) and hippocampus (Evans et al., 2018; MacQueen and Frodl, 2011; Savitz and Drevets, 2009), with some studies also noting changes in the anterior cingulate cortex, striatum, and amygdala (Gerhard et al., 2016; Gujral et al., 2017). Postmortem analyses have shown decreased neuronal soma size and reduced glial numbers in the PFC of MDD patients (Rajkowska and Stockmeier, 2013; Sanacora et al., 2008). Similarly, preclinical studies in rodents and nonhuman primates have demonstrated neuron atrophy in the hippocampus and PFC under chronic stress conditions (Duman et al., 2016; McEwen et al., 2015). Collectively, these findings indicate that depression induces structural and functional alterations in specific brain regions. Two primary hypotheses, i.e., the neuroplasticity and neurogenesis hypotheses, have been proposed at the molecular and cellular level to explain these findings (Boku et al., 2018; Price and Duman, 2020). They proposed that the pathophysiology of depression might be linked to impairments in neuroplasticity, such as neural atrophy, neural apoptosis, deficits in functional neurocircuitry, reduced generation of new neurons, and dysregulation of synaptic plasticity in cortical and limbic regions.

Neuroplasticity can be broadly defined as the capacity of the nervous system to reorganize its structure, function, and connections in response to both intrinsic and extrinsic stimuli (Cramer et al., 2011). It involves the growth and adaptability of neural pathways and synapses at the structural level (Magee and Grienberger, 2020). Functionally, this adaptability is evidenced by increased long-term potentiation (LTP), a key form of synaptic plasticity that underpins the brain’s capacity to adapt through learning and memory to the ever-changing environment. Neurogenesis, a specific form of neuroplasticity, involves the generation of new neurons in the adult brain from pluripotent stem cells (Otte et al., 2016). There is evidence that neurogenesis occurs in the dentate gyrus (DG) of adult humans (Eriksson et al., 1998; Spalding et al., 2013), suggesting that the human hippocampus retains the ability to generate neurons during adulthood, which is likely to contribute to learning and memory (Malhi and Mann, 2018). The neuroplasticity process is influenced by a variety of signaling mechanisms and molecules, including but not limited to neurotrophic factors, growth factors, cytokines, and neurotransmitters (Gonçalves et al., 2016).

Neuroplasticity has been identified as a promising target for managing depression and other neuropsychiatric disorders. The exploration of specific signaling pathways, neurotransmitters (Kraus et al., 2017), and neurotrophic factors (Castrén and Monteggia, 2021) that influence neuroplasticity in specific brain regions, such as the cortical and limbic systems, has garnered significant attention. Encouraging findings from both animal and human studies indicate that novel rapid-acting antidepressants (i.e., ketamine) (Aleksandrova and Phillips, 2021), physical exercise (Vivar et al., 2012), or learning (Sagi et al., 2012) can enhance neuroplasticity, prompting further exploration and clinical application of promising neuroplasticity-based therapies. There is compelling evidence supporting the clinical efficacy of both manual acupuncture and electroacupuncture (EA) as complementary and integrative therapies for depression (Yang et al., 2022). Extensive preclinical and clinical research has been conducted in recent years on the molecular and cellular mechanisms of acupuncture for treating depression. However, much of this research remains descriptive. To date, detailed explanations of the potential antidepressant mechanisms linked to neuroplasticity have yet to be articulated and summarized.

**Figure 1:**
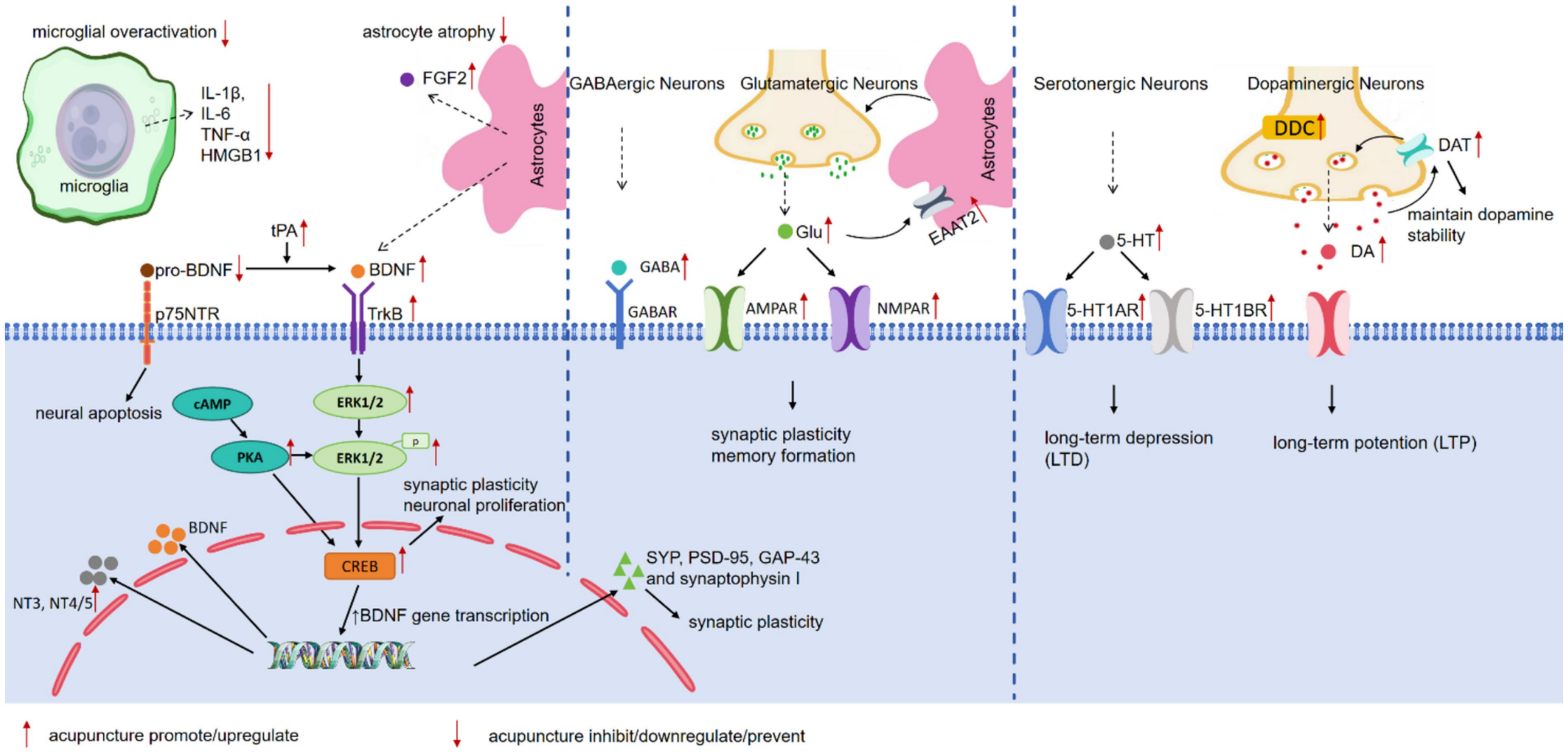
The neuroplasticity related mechanisms of acupuncture in treating depression.

To elucidate the neurobiological mechanisms associated with neuroplasticity that underpin the antidepressant effects of acupuncture on depression and to establish a solid foundation for future research, this review compiles recent data from basic research closely related to the effects of acupuncture on neuroplasticity in the treatment of depression. These studies demonstrated findings through methods such as Nissl staining, Golgi staining, transmission electron microscopy, immunohistochemical analysis, and electrophysiological recording, showing an increase in neuron numbers, the reversal of synaptic ultrastructural pathology, or the successful induction of long-term potentiation (LTP) in specific brain regions. Tables 1–2 summarizes these studies, highlighting the neuroplastic effects and related mechanisms of acupuncture treatment of depression (Figure 1), and provides a framework at the molecular and cellular levels to enhance understanding of the neuroplastic mechanisms involved in acupuncture treatment of depression.

**Table 1:** Summary for animal intervention strategy of acupuncture in treating depression.

References	Animal type	Model	Acupuncture type/retention time	Acupoints	Treatment duration
Cai et al. (2023)	Rat	CUMS	EA (2 Hz, 1 mA), 30 min	GV20, Ex-HN3	Once daily, for 2 weeks
Chen et al. (2020b)	Rat	CUMS	EA (2 Hz, 0.6 mA), 30 min	GV20, Ex-HN3	Once daily, for 2 weeks
Chen et al. (2023)	Rat	CRS	AC, 20 min	GV20, Ex-HN3	Once daily, for 28 days
Cheng et al. (2021)	Rat	CUMS	AC, 20 min	GV23, GV16	Once every other day for 14 times in total
Dai et al. (2010)	Rat	repeated stress stimulation	EA (0.6 mA, 2 Hz), 20 min	GV20, Ex-HN3	Once a day for 21 times in total
Dong et al. (2018)	Rat	CRS	AC, 20 min	GV20, Ex-HN3, SP6	Once a day for 28 times in total
Dong et al. (2021)	Rat	MCAO + CUMS	EA (2 Hz, 1 mA), 30 min	GV20, Ex-HN3, CV12, CV4	Once daily, for 3 weeks
Duan et al. (2016)	Rat	CUMS	EA (0.6 mA, 2 Hz), 30 min	GV20, Ex-HN3	Once daily, for 14 days and 28 days, respectively
Gao et al. (2021)	Rat	CUMS	EA (0.2 mA, 2 or 100 Hz), 30 min	GV20, Ex-HN3, LI4, LR3	Once daily, for 2 weeks
Gao et al. (2022)	Rat	CUMS	EA (1–1.2 mA, 2 or 100 Hz), 20 min	GV20, Ex-HN3	Once daily, for 2 weeks
Han et al. (2018)	Rat	WKY	EA (2 Hz, 0.1-4 mA), 15 min	GV20, Ex-HN3	Once daily for 3 weeks
Ji et al. (2013)	Rat	CUMS	EA (1 mA, 2 Hz), 20 min	GV20, Ex-HN3	20 min once daily for 3 weeks
Jiang et al. (2017)	Rat	CUMS	AC, 10 min	GV20, Ex-HN3	10 min per session, 1 session daily for 21 days
Jiang et al. (2020)	Rat	CUMS	EA (frequency 2 and 20 Hz), 30 min	LI4, LR3	30 min each time, 2 days per time, for a total of 21 days
Kang et al. (2021)	Rat	MCAO + CUMS	EA (disperse-dense wave, 2–20 Hz), 30 min	LI4, LR3	A total of 21 days at 30 min/session/day
Kawanokuchi et al. (2021)	Rat	SDS	AC, 20 min	GV20, Ex-HN3	10 days out of 2 weeks (20 min daily, Monday through Friday)
Lee et al. (2019)	Mice	CRS	AC, needles were turned at a rate of two spins per second for 30 s, then removed	KI10, LR8, LU8, LR4	Once daily, for 7 or 14 days
Li et al. (2017)	Rat	CUMS	EA (0.6 mA, 2 Hz), 20 min	GV20, Ex-HN3	Once every other day for three-weeks
Li et al. (2021b)	Rat	CUMS	EA (2 Hz, 1-3 mA), 30 min	PC6, SP6	Once daily for 3 weeks
Li et al. (2021a)	Rat	CUMS	AC, 10 min	GV20, Ex-HN3	6 continuous days per week for 6 weeks
Liang et al. (2012)	Rat	CUS	AC, 20 min	GV20, Ex-HN3, PC 6	Once every other day for 28 d
Lin et al. (2023)	Mice	CUMS	EA (2 Hz, 0.5 mA), 20 min	ST36	Once daily for 4 weeks
Liu et al. (2011)	Rat	CUS	EA (60 Hz for 5 s and 4 Hz for 2.5 s alternately, ≤1 mA), 30 min	GV20, EX17	Once per day for 3 consecutive weeks
Liu et al. (2023)	Rat	CUMS	AC, 20 min	GV20, EX-HN3	Once daily for 21 d
Lu et al. (2013)	Rat	CUMS	AC, 10 min	GV20, PC6	Once every other day for 4 weeks
Luo et al. (2017)	Rat	CUMS	EA (2 Hz), 30 min	LI4, LR3	5 continuous days per week for 4 weeks
Luo et al. (2020a)	Rat	CUMS	EA (2 Hz, 2 mA), 20 min	GV20, Ex-HN3	Once daily for 28 d
Pang et al. (2023)	Rat	CUMS	EA (2 Hz), 20 min	GV20, Ex-HN3, BL18	6 consecutive days per week for 3 weeks
Park and Lim (2019)	Rat	MS	AC, needles were twisted at the speed of twice a second for 30 s, then removed	ST36	Once daily for 9 d
She et al. (2015)	Rat	WKY	EA (0.1 mA - 3 mA, 2 Hz), 15 min	GV20, Ex-HN3	Once daily for 3 weeks
Sun et al. (2019)	Rat	MCAO + CUMS	AC, 40 min	GV20, GV26, CV24, GV14	6 days per week for 4 weeks
Sun et al. (2019c)	Rat	CRS	AC, 20 min	GV20, Ex-HN3, SP6	Once daily for 28 days
Sun et al. (2020)	Rat	PSD: MCAO + CUMS	AC, 40 min	GV14, GV26, GV20, GV24	6 days per week for 4 weeks
Sun et al. (2022)	Rat	MCAO + CUMS	AC, 40 min	GV14, GV26, GV20, GV24	6 days per week for 4 weeks
Tong et al. (2023)	Rat	CUMS	AC, 20 min	GV23, GV16	Every other day for four weeks
Yang et al. (2013)	Rat	CUS	EA (0.3 mA, 2 or 100 Hz), 30 min	GV20, GB34	Once daily for 4 weeks
Yang et al. (2014)	Rat	CUS	EA (0.3 mA, 2 or 100 Hz), 30 min	GV20, GB34	Once every other day for 15 consecutive days
Yao et al. (2021)	Rat	CUMS	EA (2 Hz, 0.6 mA), 30 min	GV20, Ex-HN3	Once daily for 14 days
Zhang et al. (2020)	Rat	CUMS	EA (1 mA, 2 Hz), 30 min	GV20, Ex-HN3	Once daily for 2 weeks
Zhang et al. (2021b)	Rat	CUMS	EA (2 Hz), 20 min	GV20, Ex-HN3, LI4, LR3	Once daily for 21 days
Zhang et al. (2023a)	Rat	CUMS	EA (0.6 mA, 2 Hz), 30 min	GV20, Ex-HN3	Every other day for 20 days

**Table 2:** Summary for the major mechanism of acupuncture.

References	Acupuncture type	Acupoints	Major mechanism	Neuroplasticity
Cai et al. (2023)	EA	GV20, Ex-HN3	Upregulate phosphorylation of DAT, activate the TAAR1/cAMP/PKA signaling pathway, and enhance synaptic transmission in the ventromedial PFC	Electrophysiology: EA improved synaptic transmission in vmPFC by upregulating spontaneous excitatory postsynaptic currents amplitude
Chen et al. (2020b)	EA	GV20, Ex-HN3	Promote the expression of 5-HT1A receptor in the hippocampus	Transmission electronic microscopy: EA improved the pathological changes in organelles and synaptic structures of hippocampal neurons
Chen et al. (2023)	AC	GV20, Ex-HN3	Alleviate stress-induced neuroinflammation mediated by HMGB1; depress the activation of HMGB1/TLR4 signaling pathway in amygdala; depress the hyperactivation of HPA axis	Immunofluorescence: Acupuncture changed the activation of microglia and astrocytes in amygdala
Cheng et al. (2021)	AC	GV23, GV16	Reduce oxidative stress products, and regulate the Nrf2/HO-1 signaling pathway to prevent neuronal apoptosis	Nissl’s Staining: acupuncture alleviate hippocampal neural injury, decrease the hippocampal nerve apoptosis
Dai et al. (2010)	EA	GV20, Ex-HN3	Reduce hippocampal apoptotic rate, downregulate hippocampal p-JNK level	Annexin V fluorescein isothiocyanate/ Propidium iodide double-staining: EA reduce the apoptotic rates of hippocampal neurons
Dong et al. (2018)	AC	GV20, Ex-HN3, SP6	Regulate the expression of GFAP in the hippocampal and PFC, and increase the content of serum IL-10	Regulate the astrocytes in the hippocampus and prefrontal cortex
Dong et al. (2021)	EA	GV20, Ex-HN3, CV12, CV4	Promote activation of the tPA/BDNF/TrkB pathway in the PFC	Immunofluorescence staining and Western blot analysis: EA prevent the PSD-induced decreased expression of tPA, mBDNF, and TrkB
Duan et al. (2016)	EA	GV20, Ex-HN3	Regulate multiple targets in the CREB signaling pathway and regulate neurotransmitters in the hippocampus	/
Gao et al. (2021)	EA	GV20, Ex-HN3, LI4, LR3	Reverse the synaptic deficits via the modulation of hyper-cholinergic tone	Golgi-Cox Staining and Spine Density Analysis: EA increased the spine density of mature and immature spines
Gao et al. (2022)	EA	GV20, Ex-HN3	Upregulate the BDNF/mTORC1 signaling pathway and synapse-associated proteins PSD95, Synapsin I, and GluR1 in the PFC	Golgi-Cox Staining: increase the density of dendrite spines and upregulate the expression of synapse-related proteins in PFC
Han et al. (2018)	EA	GV20, Ex-HN3	Reverse the impairment in the hippocampal CA1 synaptic plasticity, downregulate the expression of 5-HTT and 5-HT1A receptor in the hippocampus CA1 region	Electrophysiological recording: long-term potentiation was evoked at Schaffer collateral-CA1 synapses in hippocampal slices *in vitro*, the fEPSP slope increased significantly
Ji et al. (2013)	EA	GV20, Ex-HN3	Upregulate hippocampal EAAT 1 and EAAT 2 expression	Nissl’s Staining: EA effectively improve the hippocampal neuronal structure
Jiang et al. (2017)	AC	GV20, Ex-HN3	Upregulate PKA/CREB signaling pathway in the hippocampus	/
Jiang et al. (2020)	EA	LI4, LR3	Upregulate the expression of AMPAR in the hippocampus, and protect neural plasticity	Nissl Staining: EA increase the number of synapses and reduce synaptic cleft in the hippocampus
Kang et al. (2021)	EA	LI4, LR3	Upregulate the expression of BDNF and its receptor TrkB in the brain	Morphological staining: increase the number of the BDNF- and TrkB-positive cells in rats
Kawanokuchi et al. (2021)	AC	GV20, Ex-HN3	Regulate the expression of neurotrophic factors in the brain	/
Lee et al. (2019)	AC	KI10, LR8, LU8, LR4	Modulate central brain 5-HT receptor expression and central brain neural activity	/
Li et al. (2017)	EA	GV20, Ex-HN3	Regulate ERK signaling pathway in the hippocampus and prefrontal cortex.	Annexin V-FITC labeling: EA decrease the apoptosis rate in hippocampal cells
Li et al. (2021b)	EA	PC6, SP6	Inhibit the c-Fos/AP-1 signaling pathway	/
Li et al. (2021a)	AC	GV20, Ex-HN3	Inhibit the activation of microglia, reducing the expression of proinflammatory cytokines, and increasing TREM2 expression in the PFC	Immunohistochemistry: acupuncture decrease the expression of microglia in the PFC
Liang et al. (2012)	AC	GV20, Ex-HN3, PC6	Upregulate BDNF expression in the PFC and hippocampus	/
Lin et al. (2023)	EA	ST36	Prevent astrocyte atrophy and preserve ezrin-astrocyte association	3D reconstruction of astrocyte: EA ameliorates the astrocytic morphology, prevents morphological atrophy of astrocytes
Liu et al. (2011)	EA	GV20, EX17	Inhibit the astrocyte atrophy in the hippocampus	GFAP immunostaining in the DG and CA3 regions: GFAP-immunoreactive astroglial cells showing fine branches were sparsely distributed in the EA group
Liu et al. (2023)	AC	GV20, EX-HN3	Downregulate the MAPK/JNK signaling in the hippocampus	/
Lu et al. (2013)	AC	GV20, PC6	Activate the ERK/CREB pathway in the hippocampus	/
Luo et al. (2017)	EA	LI4, LR3	Enhance glial glutamate transporter EAAT2 in the hippocampus and PFC	Immunohistochemistry: EAAT2-positive cell number and protein expression in the hippocampus and prefrontal cortex were increased
Luo et al. (2020a)	EA	GV20, Ex-HN3	Upregulate the tPA/BDNF/TrkB pathway in the hippocampus	HE staining: the hippocampal neurons in the EA groups were arranged neatly, with rich layers and complete cell structures
Pang et al. (2023)	EA	GV20, Ex-HN3, BL18	Downregulate P2X7R/NLRP3/IL-1β signaling pathway in the PFC	Transmission Electron Microscopy + Nissl Staining: EA on the PFC: Cell edema was slightly alleviated, microglia were oval-shaped; the number of Nissl bodies in the EA group was higher than in the control group
Park and Lim (2019)	AC	ST36	Increase cell proliferation and enhance 5-HT synthesis	Immunohistochemistry: increase cell proliferation in the hippocampal dentate gyrus
She et al. (2015)	EA	GV20, Ex-HN3	Reverse hippocampal LTP impairment by restoring GluN2B protein expression	Electrophysiological recording: for the induction of the hippocampal Schaffer collateral-CA1 LTP, the fEPSP slope was up to 122.2872.58% in the EA groups
Sun et al. (2019)	AC	GV20, GV26, CV24, GV14	Repair hippocampal neuronal damage, which is probably related to the contents of hippocampal monoamine neurotransmitters	Transmission Electron Microscopy: alleviate the damage of the ultrastructure of hippocampal CA 1 neurons
Sun et al. (2019c)	AC	GV20, Ex-HN3, SP6	Inhibit the chronic psychological stress-hippocampal oxidative stress-mitochondrial apoptotic pathway	/
Sun et al. (2020)	AC	GV14, GV26, GV20, GV24	Activate PI3K/Akt/mTOR signaling pathway and inhibit hippocampal neuron autophagy	/
Sun et al. (2022)	AC	GV14, GV26, GV20, GV24	Upregulate the CREB/BDNF/TrkB signaling pathway in hippocampal CA1 area	/
Tong et al. (2023)	AC	GV23, GV16	Upregulate the CREB/BDNF/TrkB pathway in LHb, preserve the proper ratio of pro-BDNF to BDNF.	Golgi staining: EA reverse the less luxuriant state of the dendritic number and length in the LHb
Yang et al. (2013)	EA	GV20, GB34	Enhance the activation of ERK signaling pathways	Immunofluorescence staining: EA improved the stem cell proliferation in the DG
Yang et al. (2014)	EA	GV20, GB34	Enhance ANPs proliferation and preserving QNPs from apoptosis in the hippocampal dentate gyrus	Immunohistochemistry + Hoechst Staining: EA upregulated the number of dividing neural progenitors in the hippocampus
Yao et al. (2021)	EA	GV20, Ex-HN3	Upregulate the expression of FGF2 in the hippocampus to maintain astrocyte homeostasis	Immunohistochemistry: EA increased GFAP protein expression and the mean optical density of GFAP-immunoreactive astrocyte
Zhang et al. (2020)	EA	GV20, Ex-HN3	Partly inhibit autophagy	Transmission electron microscope: decrease the number and size of autolysosomes in hippocampus CA1 neurons
Zhang et al. (2021b)	EA	GV20, Ex-HN3, LI4, LR3	Regulate the GluN2B/CaMKII/CREB signaling pathway	Golgi and Nissl staining: reverse the decrease of the dendritic spine densities and neuron numbers in the hippocampus
Zhang et al. (2023a)	EA	GV20, Ex-HN3	Reverse the CUMS-induced decline in PNN expression, the functional impairment of GABA neurons, and regulate the excitatory synaptic proteins expression	/

5-HT, 5-hydroxytryptamine; AC, manual acupuncture; ACC, anterior cingulate cortex; Ach, Acetylcholine; AChE, acetylcholinesterase; Acupoints: Shangxing (GV23), Fengfu (GV16), Baihui (GV20), Yintang (EX-HN3), Dazhui (GV14), Shuigou (GV26), Shenting (GV24), Shangxing (GV23), Neiguan (PC6), Sanyinjiao (SP6), Hegu (LI4), Taichong (LR3), Zhongwan (CV12), Guanyuan (CV4), Zusanli (ST36), Yanglingquan (GB34), Yingu (KI10), Ququan (LR8), Jingqu (LU8), Zhongfeng (LR4), BL18, Ganshu; EX17, Anmian; ANPs, amplifying neural progenitors; AIF, apoptosis inducing factor; Akt, protein kinase B; BDNF, brain-derived neurotrophic factor; BrdU, 5-bromo-2-deoxyuridine; bFGF, basic fibroblast growth factor; caspase-3, cysteine-containing aspartate-specific proteases-3; CaMKII, calmodulin-dependent protein kinase II; cAMP, cyclic adenosine monophosphate; CRS, chronic restraint stress; CUS, chronic unpredictable stress; CUMS, chronic unpredictable mild stress; CUMS, chronic unpredictable mild stress; CREB, cAMP-response element binding protein; DDC, aromatic-L-amino-acid decarboxylase; CRS, chronic restraint stress; DG, dentate gyrus; DR, dorsal raphe; EA, electroacupuncture; EAAT2, excitatory amino acid transporter 2; FGF2, fibroblast growth factor; fEPSP, field excitatory postsynaptic potentials recording; GABA, gamma-aminobutyric acid; GAD67, γ-aminobutyric acid decarboxylase 67; GAP-43, growth-associated protein-43; GFAP, glial fibrillary acidic protein; GluR1, glutamate receptor 1; GluR2, glutamate receptor 2; HO-1, Heme oxygenase-1; IL-10, interleukin 10; LC3, light chain 3; LHb, Lateral habenular; i.p., intraperitoneally; MCAO, middle cerebral artery occlusion; MAP-2, microtubule-associated protein 2; MRN, middle raphe nucleus; MS, maternal separation; mTOR, mammalian target of rapamycin; NPY, neuropeptide Y; NT-3, neurotrophin-3; NGF, nerve growth factor; Nrf2, Nuclear factor E2-related factor 2; OVX, ovariectomized rats; PI3K, phosphatidylinositol 3-kinase; PSD-95, postsynaptic density protein-95; PFC, prefrontal cortex; Pick1, the protein interacting with C kinase 1; PKA, protein kinase A; PSD-95, postsynaptic density protein-95; p-CREB, phospho-CAMP-response element-binding; QNPs, quiescent neural progenitor; ROS, the reactive oxygen species; SDS, social defeat stress; SYP, synaptophysin; TAAR1, trace amine associated receptor 1; TEM, transmission electron microscopy; TrkB, tropomyosin receptor kinase B. WKY, Wistar Kyoto depressive model.

## 2 Targets for action of acupuncture

### 2.1 Targeting neurotrophins and their receptors

BDNF is a neuropeptide synthesized primarily in the cell bodies of neurons and glia, with the highest expression in the hippocampus, cerebellum, and cerebral cortex (Colucci-D’Amato et al., 2020). BDNF signaling critically modulating synaptic plasticity, neuronal survival, and differentiation, plays a pivotal role in the central nervous system (CNS). BDNF binds with high affinity to the tyrosine kinase B (TrkB) receptor, triggering the activation of TrkB and its downstream signaling cascades, including the mitogen-activated protein kinase (MAPK–ERK), phosphoinositide 3-kinase (PI3K-Akt), and phospholipase-C gamma (PLC-*γ*) pathways (Wang et al., 2022). These pathways are essential for supporting neuronal survival, function, and synaptic plasticity.

Preclinical research conducted by Liang et al. (2012) in animal models of depression highlighted the beneficial effects of acupuncture in upregulating BDNF mRNA and protein expression levels in the hippocampus and PFC. Additionally, studies by Sun et al. (2022a) reported that manual acupuncture stimulation increases blood BDNF concentrations, as well as BDNF protein and mRNA expression in the hippocampal CA1 area, in a poststroke depression (PSD) rat model. Kawanokuchi et al. (2021a) demonstrated that the neuroplasticity mechanism underlying acupuncture stimulation could be related to its regulatory effects on the expression of neurotrophic factors. In a social defeat stress rat model of depression, acupuncture was found to restore BDNF, neurotrophin (NT)-3, and NT-4/5 production while simultaneously suppressing nerve growth factor expression in the brain.

Cyclic AMP response-binding protein (CREB) is a transcription factor (Zhang et al., 2005) that activates the transcription of various target genes and serves as a critical regulator of BDNF-related gene expression (Finkbeiner et al., 1997). The intracellular TrkB signaling cascade facilitates CREB activation (Zarneshan et al., 2022), which in turn enhances BDNF gene transcription and promotes BDNF expression (Esvald et al., 2020), playing a pivotal role in LTP and synaptic plasticity (Tartt et al., 2022). Tong et al. (2023a) demonstrated that acupuncture may exert antidepressant-like effects by activating the BDNF/TrkB/CREB signaling pathway and decreasing the expression of pro-BDNF in the lateral habenula (LHb). Additionally, acupuncture increases the number of BDNF- and TrkB-positive cells in the brains of PSD rats (Kang et al., 2021a). Tissue plasminogen activator (tPA) regulates the balance between BDNF and pro-BDNF through activating the extracellular protease plasmin (Lee et al., 2001; Liang et al., 2018; Nagappan et al., 2009), which is part of the cleavage pathway that converts pro-BDNF to BDNF. Studies suggest that the tPA/BDNF/TrkB signaling pathway may contribute to the neuroplastic effects of EA on rats by increasing BDNF levels in the hippocampus and PFC (Dong et al., 2021; Luo et al., 2020b) and in the serum. The HE staining results corroborated these findings, showing changes in the morphology and quantity of neurons in the hippocampal CA3 area. However, the study (Luo et al., 2020) also noted lower BDNF content in the raphe nuclei, suggesting differential targeting across brain regions, which warrants further exploration and reflection.

The MAPK cascade, which includes the extracellular signal-regulated kinase (ERK) pathway, is a critical signal transduction pathway activated downstream of the BDNF-activated TrkB receptor (Sweatt, 2004). ERK plays a key role in regulating cellular fates such as growth, proliferation, differentiation, and survival (Ryu et al., 2015; Wang and Mao, 2019). The antidepressant effects of EA may be related to alterations in the extracellular microenvironment of hippocampal neural stem cells (NSCs) and the activation of ERK signaling pathways, which also contribute to the beneficial effects on promoting NSC proliferation (Yang et al., 2013). ERK1/2 is one of several ERK isoforms that has been thoroughly investigated. Research indicates that acupuncture upregulates the expression of p-ERK1/2 and BDNF in the PFC in a depression rat model (Li et al., 2018). The transcription factor CREB serves as a downstream target of ERK. Chronic stress reduces ERK and CREB phosphorylation (activation) in the rat hippocampus and PFC (Qi et al., 2008). Lu et al. (2013) demonstrated that acupuncture increases the level of p-ERK1/2 in the hippocampus and PFC and enhances p-CREB levels in the hippocampus in a depression rat model, suggesting that the ERK-CREB pathway is upregulated in the hippocampus. Additionally, the protein kinase A (PKA)/CREB signaling pathway, which regulates synaptic plasticity and learning memory, involves PKA as the upstream activator of CREB (Hu et al., 2012; Shaywitz and Greenberg, 1999). Jiang et al. (2017) reported that acupuncture upregulates PKA-*α* and p-CREB expression in the hippocampus, indicating that the mechanisms underlying the antidepressant effect of acupuncture may be related to the regulation of the ERK-CREB and PKA/CREB signaling pathway in the hippocampus.

The c-Jun N-terminal kinases (JNKs), a critical member of the MAPK family (Zeke et al., 2016), mediate cellular responses to a range of abiotic and biotic stressors (de Los Reyes Corrales et al., 2021). The JNK pathway is activated in response to various stress events, such as infection, inflammation, or oxidative stress. Research indicates that JNK signaling activation occurs in animal models of depression (Adzic et al., 2009; Egeland et al., 2015; Rogatsky et al., 1998) and that inhibition of the JNK pathway leads to increased neurogenesis and alleviation of depressive and anxiety-like behaviors (Mohammad et al., 2018). In support of this model, Liu et al. (2023a) reported that acupuncture downregulates the protein expression levels of c-JUN and p-JNK in the hippocampal CA1, CA3, and DG regions, suggesting that, at least in part, acupuncture exerts an antidepressant effect through regulating MAPK/JNK signaling.

### 2.2 Targeting neurotransmitters

#### 2.2.1 Targeting monoaminergic systems

Serotonin (5-hydroxytryptamine, 5-HT) is a monoamine neurotransmitter that has diverse functions (Sirek and Sirek, 1970). The differential physiological effects of 5-HT are mediated by the activation of any of the 15 different serotoninergic receptors, which are categorized into 7 different classes (5-HT1 to 5-HT7) (Hoyer et al., 2002). In the CNS, serotonin is primarily synthesized locally in the raphe nucleus (David and Gardier, 2016), especially the dorsal raphe (DR) (Mahar et al., 2014). Although only a minute proportion of the body’s 5-HT (approximately 5%) is found in the mature mammalian brain (Fouquet et al., 2019), it significantly influences neuronal networks during development and modulates various critical neuronal functions (Lesch and Waider, 2012), such as perception, cognitive activities and emotional responses, particularly mood regulation in the mature brain. Serotonin remains a critical neurotransmitter in the CNS with notable neuroplastic capabilities (Kraus et al., 2017), which mainly attributed to interactions between serotonergic receptors and neurotrophic proteins (Mattson et al., 2004), intracellular signaling cascades (Rantamäki and Castrén, 2008) involved in cytoskeletal rearrangement, and the modulation of cell adhesion molecules and glutamatergic transmission (Dalva et al., 2007; Sanacora et al., 2012). The dopamine (DA) system is integral to many aspects of brain function, including locomotion, affect, and cognition (Grace, 2016). Both DA and 5-HT are critical neuromodulators of synaptic plasticity but often play antagonistic roles during reward-driven learning (Wert-Carvajal et al., 2022). For example, DA enhances the induction of LTP in the hippocampus (Broussard et al., 2016; Brzosko et al., 2015). In contrast, 5-HT has been shown to induce long-term depression (LTD) in certain receptor-specific regions of the hippocampus (Kemp and Manahan-Vaughan, 2004; Lecouflet et al., 2021).

Serotonergic imbalance plays a critical role in the pathogenesis of MDD. Aversive external stimuli, such as stress, cause excessive activation of the endocrine immune system (Beurel et al., 2020). The resulting inflammatory response may trigger depression in susceptible individuals by reducing plasma tryptophan levels and diminishing brain serotonin activity (Cowen and Browning, 2015; Wichers et al., 2005). Chronic stress significantly attenuates 5-HT neurotransmission and 5-HT1A autoreceptor sensitivity (Mahar et al., 2014). Additionally, dysfunction in the DA system has also been implicated in the pathophysiology of depression. Preclinical studies using chronic unpredictable mild stress (CUMS) (Chang and Grace, 2014) or learned helplessness models (Belujon and Grace, 2014) of depression have revealed a reduction in the number of spontaneously firing DAergic neurons in the ventral tegmental area, indicating that chronic stress induces plastic changes that diminish the activity of the DAergic neuron population.

Acupuncture has been demonstrated to increase the levels of 5-HT, norepinephrine (NE), and dopamine (DA) in the hippocampus (Sun et al., 2019a), which helps to repair hippocampal neuronal damage and restore neuronal plasticity in PSD rats. One study highlighted that the antidepressant mechanism of EA may be linked to the promotion of 5-HT1A receptor mRNA and protein expression in the hippocampus (Chen et al., 2020b), thereby reversing pathological changes in hippocampal neurons. Additionally, acupuncture has been shown to enhance the expression of the 5-HT1A receptor in the cortex, hippocampus, thalamus, and hypothalamus and the 5-HT1B receptor in the cortex and thalamus (Lee et al., 2019). This provides further evidence that acupuncture can ameliorate alterations in the 5-HT system associated with depression. Acupuncture also promotes cell proliferation in the dentate gyrus of the hippocampus and increases 5-HT levels in the dorsal raphe (Park and Lim, 2019). However, one study presents different view point, it suggested that EA could alleviate depressive-like behaviors by reversing impairment in synaptic plasticity within the hippocampal CA1 region in Wistar Kyoto (WKY) depressive model rats, predominantly through the downregulation of serotonin transporter (5-HTT) and 5-HT1A receptor levels (Han et al., 2018a). Emerging evidence indicates abnormalities in the function of monoamine receptors and transporters in the brain of WKY rat (El Mansari et al., 2023). Consequently, this study holds limited significance regarding changes in 5-HT-related indicators.

Furthermore, research on dopamine has shown that EA upregulates dopaminergic signaling in the PFC, as evidenced by increased expression of a critical enzyme in this pathway, aromatic L-amino acid decarboxylase (DDC) (Zhang et al., 2021a). Studies have also shown that EA promotes the activation of the dopamine transporter (DAT), which plays a crucial role in maintaining dopamine stability in the synaptic cleft, and improves synaptic transmission in the ventromedial PFC by upregulating spontaneous excitatory postsynaptic current amplitude (Cai et al., 2023). The potential mechanism for these effects may be related to the activation of the trace amine-associated receptor 1 (TAAR1)/cyclic adenosine monophosphate (cAMP)/protein kinase A (PKA) signaling pathway, as there is evidence that TAAR1 activation has antidepressant potential and can modulate DAT function or quantity through TAAR1 signaling (Espinoza et al., 2018; Rutigliano and Zucchi, 2020).

#### 2.2.2 Targeting glutamatergic systems

Glutamate (Glu) serves as the primary excitatory neurotransmitter in the CNS (Duman et al., 2019) and is indispensable for a broad range of behaviors, including learning and memory, emotional responses, sensory input and integration, and motor system activity (Mattson et al., 2018). Through its specific receptor subtypes, namely, N-methyl-d-aspartate receptors (NMDARs) and α-amino-3-hydroxy-5-methyl-4-isoxazole propionic acid receptors (AMPARs), glutamate plays a crucial role in regulating synaptic and neuronal plasticity, which in turn influences fundamental human processes such as mood, cognition, learning, and reward (Murrough et al., 2017). Under normal conditions, moderate levels of NMDA receptor activation are beneficial, promoting neuroprotective signaling pathways. This includes the activation of the RAS-mitogen-activated protein kinase (RAS-MAPK) pathway and CREB-mediated induction of survival gene (Krishnan and Nestler, 2008). Under pathological conditions, however, glutamate-mediated excitotoxicity via the action of extrasynaptic NMDARs is known to be a potent neuronal excitotoxin, inducing either rapid or delayed neurotoxicity (Sanacora et al., 2008).

Clinical studies employing *in vivo* proton magnetic resonance spectroscopy (MRS) have consistently revealed a reduction in glutamate metabolite concentrations in the medial prefrontal cortex (mPFC) (Moriguchi et al., 2019) of patients with depression. This finding is supported by postmortem studies that reported alterations in glutamate receptor subtypes within PFC subregions of depressed subjects compared to controls (Feyissa et al., 2009; Gray et al., 2015), highlighting abnormalities in glutamatergic transmission in major depression patients. Additionally, morphological studies have revealed that chronic stress reduces the structure and function of glutamate pyramidal neurons in the mPFC and hippocampus, providing further evidence of glutamate neuronal atrophy in these regions.

Preclinical research has indicated that CUMS for 28 days leads to significant decreases in the levels of 5-HT, Glu, and gamma-aminobutyric acid (GABA) in the hippocampus. This finding is consistent with findings from ^13^C-MRS studies (Banasr et al., 2010) demonstrating that chronic stress exposure reduces the cycling and metabolism of glutamate and glutamine, as well as GABA, in the PFC of rats. EA for 14 days has been shown to reverse this trend by increasing the levels of these neurotransmitters in the hippocampus (Duan et al., 2016). In the context of CUMS, EA reportedly enhances the expression of the glial glutamate transporter EAAT2 in both the hippocampus and PFC, alleviating depressive-like behaviors (Luo et al., 2017a). Moreover, EA has been validated to increase the expression of AMPA glutamate receptor (AMPAR) and AMPAR-related proteins, such as glutamate receptor 1 (GluR1), glutamate receptor 2 (GluR2), and Stargazin, and protein interacting with C kinase 1 (Pick1) in the hippocampus. These modifications in synaptic strength and synaptic responses significantly impact synaptic plasticity (Lu et al., 2009; Tomita et al., 2005). EA also promotes neural plasticity by upregulating synapse-related proteins, including synaptophysin (SYN), postsynaptic density protein-95 (PSD-95), and growth-associated protein-43 (GAP-43), in the hippocampus (Jiang et al., 2020). The neuroplasticity effects of EA were confirmed by Nissl staining, which revealed an increase in synapses and a reduction in synaptic clefts in the hippocampus. The functional properties of NMDARs are determined by their subunit composition (Cull-Candy et al., 2001). At synaptic sites, NMDARs typically contain GluN2A subunits, which mediate long-term synaptic plasticity. In contrast, extra-synaptic NMDARs are generally enriched in GluN2B subunits, and are thought to constitute a major signaling pathway that triggers neuronal death (Tian et al., 2021). Studies have suggested (Zhang et al., 2021b) that EA ameliorates depression-like behaviors, potentially through its influence on synaptic plasticity by reducing GluN2B levels. This reduction may inhibit the overactivation of GluN2B-containing NMDARs and increase the expression of synaptic plasticity-related proteins including microtubule-associated protein 2 (MAP-2), PSD-95, and SYN in the hippocampus. Additionally, there is evidence that EA increased NR2A expression level as well as decreased NR2B expression level in the hippocampus (Guo et al., 2021), leading to an affirmative conclusion that EA promotes synaptic plasticity in the hippocampus of depression-modeled rats. Research examining the effect of acupuncture on glutamate levels in the brain has yielded mixed results. In contrast to the previous finding, one study suggest that EA decreases hippocampal glutamate levels, leading to varying conclusions that necessitate further verification (Guo et al., 2021). We carefully reviewed the protocol in these two articles (Duan et al., 2016; Guo et al., 2021) and found that both studies used male Sprague–Dawley rats for a four-week CUMS modeling process and utilized high-performance liquid chromatography (HPLC) to detect hippocampal neurotransmitters, with no notable methodological differences. However, the modeling outcomes of these two studies vary significantly: one study revealed a decrease in glutamate levels due to CUMS, whereas the other reported an increase. We found through literature review that there is considerable diversity in the early findings reported alterations of glutamate in blood, CSF, and brain tissue. Acute stress increases extracellular glutamate in the mPFC and hippocampus, while studies demonstrate that chronic stress exposure decreases the cycling and metabolism of glutamate and glutaminevin rat PFC (Duman et al., 2019). The discrepancies in the results observed between these two studies may be due to variations in the intensity of the CUMS modeling process. Future research endeavors should meticulously consider this factor, ensuring uniformity and comparability in the modeling intensity across studies to enable the derivation of more precise and dependable conclusions. Furthermore, it is notable that, emerging evidence shows deficits in glutamatergic signaling in the brain of WKY rat (Millard et al., 2020). Future experiments should carefully consider the suitability of using this variety of mice for the relevant research.

### 2.3 Targeting glia

#### 2.3.1 Targeting astrocytes

Astrocytes are abundant (Freeman, 2010) and intricately structured glial cells in the central nervous system (CNS) (Oberheim et al., 2009; Stogsdill et al., 2017). They form extensive contacts with other brain cells and perform diverse functions, including ion and neurotransmitter homeostasis, synapse formation, modulation, function and elimination (Endo et al., 2022). Astrocytes are closely associated with synapses, as they infiltrate the neuropil via numerous cellular processes to interact with thousands of synapses and actively participate in synapse formation and plasticity through various secreted and contact-mediated signals (Clarke and Barres, 2013; Stogsdill et al., 2017). They also play a crucial role in regulating glutamate neurotransmission (Sanacora and Banasr, 2013) by actively regulating the uptake, metabolism, and recycling of glutamate (Rajkowska and Stockmeier, 2013), thus preventing synaptic spillover and excitotoxicity (Lener et al., 2017).

Emerging evidence suggests that the neuropathology of MDD is characterized by notable reductions in astrocyte density and the expression of astrocyte markers without evident neuronal loss in both patients with MDD and depressive model mice (Rajkowska and Stockmeier, 2013). Postmortem studies have reported a decrease in glial cell density and number, specifically in certain frontal-limbic regions, in subjects with MDD but not in senior citizens (Bowley et al., 2002; Cotter et al., 2002; Gittins and Harrison, 2011; Khundakar et al., 2011a; Khundakar et al., 2011b; Ongür et al., 1998; Rajkowska et al., 1999). Reductions in the protein and mRNA expression of astrocyte markers, particularly glial fibrillary acidic protein (GFAP), as indicated by several preclinical studies and postmortem studies (Araya-Callís et al., 2012; Banasr et al., 2010; Bernard et al., 2011; Chandley et al., 2013; Choudary et al., 2005; Ernst et al., 2011; Johnston-Wilson et al., 2000; Klempan et al., 2009; Sequeira et al., 2009; Si et al., 2004; Sun et al., 2012; Webster et al., 2005), suggest a significant reduction in the number of astrocytes. Moreover, stress leads to diminished secretion of neurotrophic factors and increased production of cytokines (Schmidt et al., 2011) by astrocytes, as well as a reduced astrocytic response to neuronal injury (Laping et al., 1994). This collectively contributes to disrupted neuroplasticity and cellular resilience (Manji et al., 2000; Pittenger and Duman, 2008) in depression.

Preclinical studies have shown that EA prevents astrocyte atrophy in the prefrontal cortex and alleviates depressive-like behavior in mice subjected to CUMS (Lin et al., 2023a), providing experimental evidence that EA enhances the presence of astrocytes in the active milieu of the brain. Fibroblast growth factor 2 (FGF2) is a pleiotropic protein involved in regulating a myriad of cellular processes, including the proliferation, differentiation, and survival of various cell types (Belov and Mohammadi, 2013; Bikfalvi et al., 1997; Krzyscik et al., 2022; Xie et al., 2020). EA has been shown to upregulate the expression of FGF2 in the hippocampus and increase both the protein expression of the astrocyte marker GFAP and the mean optical density of GFAP-immunoreactive astrocytes (Yao et al., 2021a). Considering that FGF2 knockdown significantly reduces astrocyte proliferation and induces astrocyte apoptosis (Yao et al., 2021a), it is suggested that EA maintains astrocyte homeostasis by modulating FGF2 expression. Additionally, animal research has demonstrated that EA enhances GFAP mRNA and protein expression in the hippocampus (Liu et al., 2011), indicating its role in preventing hippocampal glial atrophy. Other studies corroborate these findings, reporting that acupuncture therapy increases hippocampal GFAP protein expression while decreasing it in the prefrontal cortex (Dong et al., 2018).

#### 2.3.2 Targeting microglia

Microglia are the principal innate immune cells that reside in the central nervous system (CNS) (Nayak et al., 2014). These cells act as crucial sentinels, maintaining CNS homeostasis and responding swiftly to damage or infection (Beurel et al., 2020). In their quiescent state, microglia secrete trophic factors that are essential for neuronal development, maintenance, and function throughout life. Their phagocytic activity is also critical, as they participate in the clearance of dead cells, as well as in synaptogenesis and synaptic pruning—processes vital for maintaining normal brain homeostasis. Even under resting conditions, the processes of microglia are highly dynamic; they perpetually scan their surroundings and communicate directly with neurons, astrocytes, and blood vessels. When faced with damage, inflammation, or other pathological changes, microglia undergo transformations, activate inflammatory functions, and initiate genetic programs designed to address and repair CNS insults.

In pathological states, abnormalities in microglia contribute significantly to the pathology of depression, primarily through the promotion of neuroinflammation (Yirmiya et al., 2015). This is often related to stress-induced activation of the sympathetic nervous system and the HPA axis (Ader et al., 1995; Won and Kim, 2016), which increase circulating glucocorticoids (de Kloet et al., 2008; Hunter et al., 2016) and subsequently activate the immune system. This activation triggers inflammatory responses from proinflammatory microglia, resulting in increased release of proinflammatory cytokines. Studies have highlighted the critical role of microglia-related neuroinflammation and neuronal atrophy in depression (Price and Duman, 2020).

Recent research has focused primarily on clarifying the mechanisms by which acupuncture reduces neuroinflammation. Li et al. (2021a) discovered that manual acupuncture therapy reversed the elevated levels of interleukin-1beta (IL-1beta), interleukin-6 (IL-6), and the microglial marker ionized calcium-binding adaptor molecule 1 (Iba-1) and decreased the gene expression of triggering receptor expressed on myeloid cells 2 (TREM2) in the PFC. These findings suggest that acupuncture mitigates neuroinflammation by inhibiting microglial overactivation, reducing the expression of proinflammatory cytokines, and enhancing TREM2 expression in the PFC. High mobility group box-1 (HMGB1) is recognized as an endogenous risk factor and initiating signal for neuroinflammation (Weber et al., 2015; Zandarashvili et al., 2013) and is actively released by microglia and neurons under CUMS conditions (Rana et al., 2021; Wang et al., 2020). Chen et al. (2022) reported that acupuncture alleviates neuroinflammation by downregulating HMGB1 expression and microglial activation in the hippocampus and reducing tumor necrosis factor-α (TNF-α) levels in the serum.

### 2.4 Other targets for neuroplasticity

Several animal studies have shown that acupuncture can prevent neuronal apoptosis or autophagy. Acupuncture intervention significantly reduces oxidative stress markers, such as reactive oxygen species (ROS) and H_2_O_2_, by upregulating the nuclear factor E2-related factor 2 (Nrf2)/haem oxygenase-1 (HO-1) signaling pathway. This pathway plays a crucial role in the cerebral antioxidant system (Suzuki and Yamamoto, 2015), thus preventing neuronal apoptosis (Cheng et al., 2021). Chronic stress results in elevated levels of ROS, which are intimately linked to oxidative stress and subsequently trigger the activation of the mitochondrial apoptosis pathway. Acupuncture effectively reduces ROS levels and the expression of key factors in the apoptosis pathway, including cytochrome C, cysteine-containing aspartate-specific protease-3 (caspase-3), and apoptosis-inducing factor (AIF) proteins, in the hippocampus (Sun et al., 2019c).

EA partially inhibits autophagy by reducing the number and size of autolysosomes and decreasing the levels of the autophagic biomarker light chain 3 (LC3) and the LC3-II/LC3-I ratio in hippocampal CA1 neurons (Zhang et al., 2020). Mammalian target of rapamycin (mTOR) is an autophagy inhibitory kinase, and its activation leads to the suppression of cellular autophagy. The phosphatidylinositol 3-kinase (PI3K)/protein kinase B (Akt)/mTOR signaling pathway plays a pivotal regulatory role in autophagy. Acupuncture promotes the activation of the PI3K/Akt/mTOR signaling pathway, thereby inhibiting hippocampal neuron autophagy. This is evidenced by decreased expression of Beclin1, LC3B-II/I, and LC3B-II in the CA1 region of the hippocampus (Sun et al., 2020). Beclin1 is a critical factor in the activation of autophagy, LC3 serves as a reliable marker of autophagosomes, and the LC3B-II/I ratio reflects the level of cellular autophagy. Furthermore, EA enhances the proliferation of amplifying neural progenitor cells (ANPs) while suppressing the apoptosis of quiescent neural progenitor cells (QNPs) in the hippocampal dentate gyrus (Yang et al., 2014).

PNNs are structures within the extracellular matrix of the central nervous system that play crucial roles in synaptic plasticity and protection against external oxidative stress (Hirono et al., 2018). Research has shown that EA enhances the expression of PNN, GABA synthetase glutamic acid decarboxylase 67 (GAD67), and excitatory synaptic proteins, including GLuR1 and PSD-95, in the mPFC of rats subjected to CUMS (Zhang et al., 2023). These findings confirm the antidepressant effects of EA on synaptic plasticity.

Further studies have demonstrated that EA increases the spine density on specific dendrites of layer V pyramidal neurons and increases the expression of synaptic proteins such as BDNF, GluR1, GluR2, PSD-95, and synaptophysin I in functional areas of the PFC (Gao et al., 2021). This finding suggested that the mechanism by which EA ameliorates depressive-like behaviors is related, at least in part, to the promotion of synaptic plasticity. Additionally, findings indicate a decrease in acetylcholine expression and an increase in acetylcholinesterase expression in the PFC, suggesting that modulation of the hypercholinergic tone may contribute to the antidepressant and neuroplastic effects of EA. This area presents new research directions and requires further exploration.

## 3 Conclusion

Growing clinical and preclinical evidence supports the effectiveness of acupuncture as a complementary and integrative therapy for depression. The modulatory effect of acupuncture may derive from both acupoint specificity and electrical stimuli, highlighting the complexity of this therapeutic method. As a traditional medical technique, acupuncture demonstrates considerable variability in acupoint selection, manipulative techniques, and stimulation intensity. Different acupuncturists may employ different acupuncture strategies, and even the same practitioner may modify the acupuncture protocol based on timing or patient response heterogeneity. This makes it a challenge to quantify the treatment. Furthermore, the current trend in clinical practice is to integrate acupuncture and mainstream medicine. In treating depression, acupuncture serve as a supplementary and alternative approach, improve symptoms of patients, and enhance the efficacy of antidepressant medications or other therapies. The aforementioned emphasizes the complexity of acupuncture and the challenges for quantify this treatment, and the requirement of more rigorous approaches to investigate the underlying mechanisms of acupuncture.

This review has summarized numerous potential targets related to neuroplasticity that contribute to the antidepressant effect of acupuncture. These targets include the modulation of neurotrophic factors and their receptors, neurotransmitters (primarily within the monoaminergic and glutamatergic system), growth factors, and glia (primarily within astrocytes and microglia). It is important to acknowledge that there are many other potential mechanisms involved in the pathophysiology of depression that were not captured in this paper but may be modulated by acupuncture interventions, such as effects on inflammation, the HPA axis, the gut microbiota, the gene expression of neuropeptides, mitochondrial biogenesis, and extracellular ATP levels. Although this review focused primarily on the interplay between acupuncture, neuroplasticity, and depression in the hippocampus and PFC, it did not fully explore the diversity of plasticity across different brain regions that vary according to brain circuits. Thus, the conclusions presented should be further substantiated with clinical research employing more advanced diagnostic tools such as diffusion tensor imaging and proton magnetic resonance spectroscopy.

## Glossary

5-HT: 5-hydroxytryptamine, Serotonin
5-HTT: The serotonin transporter
AIF: Apoptosis-inducing factor
Akt: Protein kinase B
AMPAR: The expression of AMPA glutamate receptor
AMPARs: α-amino-3-hydroxy-5-methyl-4-isoxazole propionic acid receptors
ANPs: The amplifying neural progenitor cells
BDNF: Brain-derived neurotrophic factor
cAMP: Cyclic adenosine monophosphate
caspase-3: Cysteine-containing aspartate-specific proteases-3
CNS: The central nervous system
CNS: Central nervous system
CREB: Cyclic AMP response-binding protein
CUMS: The chronic unpredictable mild stress
DA: Dopamine
DAT: The dopamine transporter
DDC: Aromatic-L-amino-acid decarboxylase
DG: The dentate gyrus
DR: The dorsal raphe
EA: Electroacupuncture
ERK: The extracellular signal-regulated kinase
FGF2: Fibroblast growth factor 2
GABA: Gamma-aminobutyric acid
GAD67: GABA synthetase glutamic acid decarboxylase 67
GAP-43: Growth-associated protein-43
GFAP: Glial fibrillary acidic protein
Glu: Glutamate
GluR1: Glutamate receptor 1
GluR2: Glutamate receptor 2
HMGB1: High mobility group box-1
HO-1: Heme oxygenase-1
HPA: Hypothalamic–pituitary–adrenal axis
Iba-1: The microglial marker Ionized calcium-binding adaptor molecule 1
IL-1beta: Interleukin-1beta
IL-6: Interleukin-6
JNKs: The c-Jun N-terminal kinases
LC3: The autophagic biomarker light chain 3
LHb: The lateral habenular
LTD: Long-term depression
LTP: Long-term potentiation
MAP-2: Microtubule-associated protein 2
MAPK–ERK: The mitogen-activated protein kinase
mPFC: The medial prefrontal cortex
MDD: Major depressive disorder
MRS: Magnetic resonance spectroscopy
mTOR: The mammalian target of rapamycin
NE: Norepinephrine
NMDARs: N-methyl-d-aspartate receptors
Nrf2: Nuclear factor E2-related factor 2
NSCs: Neural stem cells
NT: Neurotrophin
PFC: The prefrontal cortex
PI3K: The phosphatidylinositol 3-kinase
PI3K-Akt: phosphoinositide 3-kinase
Pick1: Protein interacting with C kinase 1
PKA: The protein kinase A
PKA: Protein kinase A
PLC-*γ*: Phospholipase-C gamma
PNN: Perineuronal nets
PSD: Poststroke depression
PSD-95: Postsynaptic density protein-95
QNPs: The quiescent neural progenitor cells
RAS-MAPK: The RAS-mitogen-activated protein kinase pathway
ROS: Reactive oxygen species
SYN: Synaptophysin
SYP: Synaptophysin
TAAR1: The trace amine-associated receptor 1
TNF-α: Tumor necrosis factor-α
tPA: Tissue plasminogen activator
TrkB: The tyrosine kinase B receptor
WKY: Wistar Kyoto
